# Immunotherapy in Malignant Pleural Mesothelioma

**DOI:** 10.3389/fonc.2020.00187

**Published:** 2020-02-21

**Authors:** Cornedine J. de Gooijer, Frank J. Borm, Arnaud Scherpereel, Paul Baas

**Affiliations:** ^1^Department of Thoracic Oncology, The Netherland Cancer Institute, Amsterdam, Netherlands; ^2^Department of Pulmonary and Thoracic Oncology, CHU, Lille, France

**Keywords:** immunotherapy, malignant pleural mesothelioma, angiogenesis inhibitors, PD-L1, dendritic cell therapy

## Abstract

The only registered systemic treatment for malignant pleural mesothelioma (MPM) is platinum based chemotherapy combined with pemetrexed, with or without bevacizumab. Immunotherapy did seem active in small phase II trials. In this review, we will highlight the most important immunotherapy-based research performed and put a focus on the future of MPM. PD-(L)1 inhibitors show response rates between 10 and 29% in phase II trials, with a wide range in progression free (PFS) and overall survival (OS). However, single agent pembrolizumab was not superior to chemotherapy (gemcitabine or vinorelbine) in the recent published PROMISE-Meso trial in pre-treated patients. In small studies with CTLA-4 inhibitors there is evidence for response in some patients, but it fails to show a better PFS and OS compared to best supportive care in a randomized study. A combination of PD-(L)1 inhibitor with CTLA-4 inhibitor seem to have a similar response as PD-(L)1 monotherapy. The first results of combining durvalumab (PD-L1 blocking) with cisplatin-pemetrexed in the first line are promising. Another immune treatment is Dendritic Cell (DC) immunotherapy, which is recently tested in mesothelioma, shows remarkable anti-tumor activity in three clinical studies. The value of single agent checkpoint inhibitors is limited in MPM. There is an urgent need for biomarkers to select the optimal candidates for immunotherapy among MPM patients in terms of efficacy and tolerance. Results of combination checkpoint inhibitors with chemotherapy are awaiting.

## Introduction

Malignant pleural mesothelioma (MPM) is a rare, aggressive malignancy with limited treatment options. Surgery is controversial since only a minority of patients is fit enough to be a surgical candidate and a complete microscopic (and sometimes macroscopic) resection is not realistic. Therefore, the indication of surgery, within a multimodal strategy, has become stricter over the last years. At this time, the only registered systemic treatment is platinum-based chemotherapy combined with pemetrexed, with or without bevacizumab. Numerous phase I and II trials have been performed to make a step forward in the treatment of MPM. Immunotherapy seemed promising in small phase II trials. However, single agent pembrolizumab was not superior to chemotherapy (gemcitabine or vinorelbine) in the recent published PROMISE-Meso trial. Currently, we are awaiting the outcome of randomized phase III studies with immunotherapy in the first line. In this review, we will highlight the most important immunotherapy-based research performed and put a focus on the future of MPM.

## PD-(L)1 Blocking

Several PD-(L)1 inhibitors have been tested in patients with progressive disease after first line chemotherapy. The KEYNOTE-028 phase I trial was the first study testing a PD-1 inhibitor (pembrolizumab) in 25 patients with a PD-L1 immunohistochemistry expression (IHC) ≥1%. The trial reported a response rate of 20%, a disease control rate (DCR) of 72% with a median duration of response of 12 months (1). Desai et al. reported similar results in 65 patients treated with pembrolizumab, in a unselected patient population (2). The response rate was 19%, a DCR of 47% and with a median progression free survival (mPFS) of 4.5 months (Table 1). Metaxas et al. reported the efficacy of this checkpoint inhibitor using real world data. In 93 patients they observed an objective response rate (ORR) of 18%. However, the mPFS was only 3.1 months with an OS of 7.2 months (3).

**Table 1:** Overview of study results.

**References**	**Agent**	**N**	**Line of treatment**	**DCR** **%**	**ORR** **%**	**mPFS *months***	**mOS *months***	**Response by PD-L1 status nr of pts and %**	**Response in subtypes nr of pts and %**	**Study type**
Alley et al. (1)	Pembro	25	>1st	72 *RECIST 1.1*	20	5.4	18.0	All patients ≥1% PDL-1	Not reported	Ib
Desai et al. (2)	Pembro	65	2nd, 3rd	66 *RECIST 1.1*	19	4.5	11.5	<1%: 2/26 (7%) 1–49%: 4/16 (25%) >50%: 6/20 (31%)	E:8/50 (16%) B:1/10 (10%) S: 2/5 (40%)	II
Metaxes et al. (3)	Pembro	93	1st, 2nd, 3rd	48 *Unknown*	18	3.1	7.2	<5%: 5/45 (11%) 5–49% 5/12 (42%) ≥ 50%: 4/9 (44%)	E: 11/67 (16%) B+S: 6/25 (24%) NE: 1	RS
Okada et al. (4)	Nivo	34	2nd, 3rd	68 *mRECIST*	29	6.1	17.3	<1%: 1/12 (8%) ≥1%: 8/20 (40%) NE: 1/2 (50%)	E: 7/27 (26%) B:1/4 (25%) S: 2/3 (67%)	II
Quispel-Janssen et al. (5)	Nivo	34	2nd, 3rd	47 *m-iRECIST*	24	2.6	11.8	(PR+SD) 0%: 8/21 (38%) 1–5%: 2/3 (67%) 5–50%: 0/2 (0%) >50%: 1/1 (100%) NE: 2/7 (29%)	E: 7/28 (25%) B: 2/4 (50%) S: 0/2 (0%)	II
Hassen et al. (6)	Ave	53	>1st	58 *RECIST 1.1*	9 1 CR	4.1	10.7	<5%: 2/27 (7%) ≥5%: 3/16 (19%)	Not reported	1b
Disselhorst et al. (7)	Nivo + ipi	34	2nd, 3rd	67 *mRECIST*	38	6.2	NR (12.7–NR)	(PR+SD) 0: 6/19 (32%) ≥1%: 11/15 (73%) ≥50% 4/5 (80%)	Not reported	II
Scherpereel et al. (8)	Nivo vs Nivo + ipi	63 vs. 62	2nd, 3rd, 4th	N: 40 NI: 52 *mRECIST*	N: 17 NI: 30	N: 4.0 NI: 5.6	N: 11.9 NI: 15.9	N: <1: 3/31 (10%) ≥1: 7/19 (37%) NE: 1/13 (8%) NI: <1: 9/27 (33%) ≥1: 7/22 (32%) NE: 3/13 (23%)	N: E:7/52 (13%) B+S: 4/11 (36%) NI: E: 15/53 (28%) B+S:3/9 (33%)	RA II
Calabro et al. (9)	Treme + durva	40	1st, 2nd	65 *mRECIST*	28	8.0	16.6	0%: 4/15 (27%) ≥1%: 7/23 (30%) NE: 2	E: 9/32 (28%) B+S:2/7 (29%)	II
Calabro et al. (10)	Treme	29	>1st	31 *RECIST*	7	6.2	10.7	Not reported	E:9/25 (36%) B: 0/1 S: 0/3	II
Calabro et al. (11)	Treme	29	2nd	52 *iRECIST* 38 *mRECIST*	14 *iRECIST* 3 *mRECIST*	6.2	11.3	Not reported	Not reported	II
Maio et al. (12)	Treme vs. placebo	571	>1st	T: 4.5 P: 1.1 *mRECIST*	T: 27.7 P: 21.7	T: 2.8 P: 2.7	T: 7.7 P: 7.3	Not reported	HR for survival event E: 0.95 (0.77-1.18) B: 1.04 (0.55-1.98) S: 0.68 (0.34-1.39)	RA IIb
Nowak et al. (13)	Durva + chemo	54	1st	48 *mRECIST* 50 *iRECIST*	*mRECIST* 48% *iRECIST* 50%	6.9	Not reported	Not reported	Not reported	II
Popat et al. (14)	Pembro vs. chemo (gemcitabine or vinorelbine)	142	2nd	Pembro 45, chemo 38 *RECIST 1.1*	P:22 C: 6	P: 2.5 C: 3.4 HR: 1.06 (0.73–1.53)	P: 10.7 C: 11.7	Pembrolizumab <1% 3/19 (16%) ≥1%: 10/32 (31%) NE: 3/22(14%) Chemotherapy <1% 1/17 (6%) ≥1%: 3 /34 (9%) NE:0 /20 (0%)	HR for survival PD-L1 <1% 1.26 (p=0.57) HR for survival PD-L1 ≥1%: 1.06 (P=0.82)	RA III

*Pembro, Pembrolizumab; Nivo, Nivolumab; Ipi, Ipilimumab; Treme, Tremelimubab; mRECIST, Modified RECIST criteria for malignant pleural mesothelioma; M-I-RECIST, Combination of modified RECIST and iRECIST; N, Nivolumab; NI, nivolumab + Ipilimumab; NE, not evaluable; NR, Not reached; E, epitheloid; B, biphasic; S, Sarcomatoid; RA, Randomized; RS, Retrospective; Ave, avelumab*.

Single agent nivolumab has been tested in 2 single arm phase II trials and in the MAPS2 trial, a randomized, non-comparative phase II study of nivolumab and nivolumab-ipilimumab. All three studies showed activity with an ORR between 15 and 29% and a DCR between 44 and 68% (4, 5, 8). In one of the phase II trials (NivoMes), the mPFS was disappointing with only 2.6 months (5). The second study tested nivolumab monotherapy (MERIT) and showed a higher mPFS of 6.1 months (4). In the combination study of the MAPS-2, the nivolumab monotherapy reported a mPFS of 4.0 months (8). The study with avelumab, a PD-L1 blocker, showed less efficacy with a response rate of 9.4% in 53 patients and a mPFS of 3.9 months (6).

The first randomized study in patients with recurrent MPM has recently been presented at the ESMO congress 2019; ETOP PROMISE-meso, randomizes patients to chemotherapy (gemcitabine or vinorelbine) vs. pembrolizumab. The primary endpoint; PFS was not met with a median PFS for pembrolizumab of 2.5 (95% CI 2.1–4.2) vs. 3.4 months (2.2–4.3) in the chemo arm, HR = 1.06 [0.73–1.53], *p* = 0.76. Surprisingly, the response rate was significantly higher in the pembrolizumab arm (22%) compared to chemotherapy (6%; *p* = 0.004), despite an equal PFS. The median OS was 10.7 months for patients in the pembrolizumab arm vs. 11.7 months for chemotherapy, HR = 1.05 ([0.66–1.67]; *p* = 0.85). Forty-five patients out of the chemotherapy arm crossed over to pembrolizumab after progression on chemotherapy. Accounting for crossover yielded a similar OS result. Treatment-related adverse events were similar in both groups. (TrAE) grade ≥3 were experienced by 19% in the pembrolizumab arm vs. 24% chemotherapy arm (14).

The CONFIRM trial in UK is ongoing, in which 336 patients with progression after at least 2 treatment lines will be randomized to 12 months treatment with nivolumab or placebo (15). The primary endpoint is OS, with secondary endpoint i.e., quality of life (QoL). These trials will hopefully provide evidence of the potential benefit of the use of PD-1 blocking in the treatment of relapsed mesothelioma.

## CTLA-4 Inhibitors

To date, only three studies were performed with an anti-cytotoxic T lymphocyte antigen 4 (CTLA-4) inhibitor alone. Initially, the phase II trials MESOT-TREM-2008 (10) and MESOT-TREM-2012 (11) trial showed some promising results and a large randomized controlled trial (DETERMINE) was initiated (12). In both MESOT-TREM trials 29 patients with MPM were included and treated with tremelimumab. In the first trial from 2008, two patients had a partial response and 7 others achieved disease control.

In the 2008 study the treatment dosage was 15 mg/kg every 90 days. After a retrospective analysis of a study in melanoma with tremelimumab, it was suggested that the dosage of tremelimumab administered was to low (16). In the subsequent MESOT-TREM-2012 trial, patients were treated with tremelimumab 10 mg/kg every 4 weeks, and after 6 cycles every 12 weeks. The response rate was slightly better, with a PR of 4 patients and disease control with a total of 15 patients, when measured with immune RECIST criteria. However, in the 2008 study, the modified RECIST criteria were used and based on these criteria only 1 patient had a partial response and 11 in total achieved disease control in the 2012 study.

Based on the results of the MESO-TREM studies, a large randomized controlled trial (DETERMINE) with higher dosage of tremelimumab was performed. Five hundred seventy-one patients were included and randomized (2:1) to tremelimumab or placebo. There were no significant differences in response or survival between the two groups. In earlier performed studies with PD-L1 blockers, a better result was suggested in the non- epitheloid subtype. The DETERMINE study did not confirm this observation. Although there seems to be a trend in the sarcomatoid group in favor of tremelimumab, the number of patients are too small to detect a significant difference. To explain the difference between de MESOT-TREM and the DETERMINE studies, one may argue that the number of patients was too small in DETERMINE trial; There were only 3 patients with a sarcomatoid subtype in this study. As known this is a more aggressive subtype and therefor faster growing. Only two patients in the study had a partial response (12).

## Combination Therapy

As seen in melanoma and NSCLC, there can be an additive or synergic effect when combining CTLA-4 with PD-(L1) checkpoint inhibitors. The non-comparative MAPS-II trial, randomizing patients between nivolumab alone or nivolumab with ipilimumab showed clinical activity in both arms with a DCR of 40 and 52%, an ORR of 19 vs. 28% and mPFS of 4.0 and 5.6 months respectively. The combination group had a slightly higher proportion of drug-related adverse events (93% with combination vs. 89% with monotherapy and 3 toxicity-related deaths (vs. none in the monotherapy group). In their study, the French investigators concluded that nivolumab monotherapy with or without ipilimumab provides a clinically meaningful response (8). Updated results showed a median OS of 11.9 months (6.7–17.4) in the nivolumab arm and 15.9 months (10.7–22.2) in the combination arm (17). The occurrence of hyper progression disease (HPD) was assessed by two formulae; Tumor Growth Rate (TGR) and Tumor Growth Kinetics (TGK). The TGK definition of HPD did impact OS after pooling data from both treatment arms. The was no significant correlation of HPD defined by TGR and OS (see Table 2).

**Table 2:** Hyper Progression Disease reported in the MAPS2 trial (17).

	**Nivolumab**	**Nivolumab + Ipilimumab**	**Both treatment arm**
**TGR**			
Number of patients with HPD	4	2	
OS			
With HPD	Mean 4.6 (0.9–7.8)	Mean 4.5 (0.5–8.6)	
Without HPD	Mean 4.0 (2.4–8.6)	Mean 5.8 (1.4–9.9)	
**TGK**			
Number of patients with HPD	7	4	
OS			
With HPD	1.6 (0.8–7.7)		
Without HPD	4.4 (2.4–10.8)		
**TGK**			
OS (months)			
With HPD (*N* = 11)			2.6 (0.8–7.7)
Disease control (*N* = 75)			23.1 (16.1–26.7)^*^
Progressive disease (*N* = 42)			5.5 (2.6–8.9)^**^

*It is not reported in how many patients Hyper Progressive Disease (HPD) could be assessed*.

* *Hazard ratio (HR, disease control vs. HPD): 0.12 (0.06–0.25; P < 0.001)*.

** *HR (progressive disease vs. HPD): 0.37 (0.19–0.75; P = 0.006)*.

*HR for correlation of OS and TGR is not reported*.

*TGR, Tumor Growth Rate; TGK, Tumor Growth Kinetics*.

The clinical activity of combination ipilimumab-nivolumab was also seen in the Dutch INITIATE trial with a response rate of 38% and a DCR of 68% at three months. However, the combination treatment was more toxic with 94% of patients experienced an adverse event. Most side effects were easily managed and no grade 5 toxicity was observed (7).

Tremelimumab, another CTLA-4 blocker was also tested with a PD-L1 blocker (durvalumab) in 40 patients (in first and second line) in the NIBIT trial. The ORR of 28% was comparable to the MAPS-2 trial with a DCR of 65%, a median PFS of 8.0 months and an OS of 16.6 months (9).

The combination of PD-1 blocking and chemotherapy is an effective first line treatment in NSCLC. The first results of combining durvalumab (PD-L1 blocking) with cisplatin-pemetrexed in the first line are hopeful. In the Australian DREAM study, a single arm phase II in 54 first line patients reported an ORR of 48% by mRECIST but a mPFS of 6.9 months only (13). The PFS at 6 months (PFS6) was 57% (90% CI 45–68%). An international world-wide phase III randomized study with this combination is planned, led by the USA and Australia.

At this moment multiple randomized studies are running or awaiting evaluation:

(1) The phase 3 Checkmate 743 study (NCT02899299) in which 600 patients have been randomized between cisplatin (or carboplatin)-pemetrexed or nivolumab-ipilimumab as first-line treatment. First results are expected beginning of 2020;

(2) The IND-227 (NCT02784171) study has been initiated to determine the value of pembrolizumab in the first line. This randomized phase II part of this study had three treatment arms: single agent pembrolizumab, cisplatin/pemetrexed, or a combination of the three agents. In the ongoing phase III part, extended to Italy, France (IFCT) and UK, the patients are randomized between cisplatin (or carboplatin)-pemetrexed plus pembrolizumab vs. the same chemotherapy alone. The estimated primary completion date is August 2020;

(3) The ETOP BEAT-meso trial (NCT03762018) in which 320 patients will be randomized between platinum-pemetrexed-bevacizumab with or without atezolizumab. The primary endpoint is PFS. First results are expected Q4, 2024.

## Dendritic Cell Therapy

Dendritic Cell (DC) immunotherapy is tested in several cancers. In mesothelioma, there are three clinical studies with DCs showing remarkable anti-tumor activity. In the first study published in 2010, autologous monocyte-derived DCs loaded with autologous tumor cell lysate were given to 9 MPM patients. The DCs were administrated in three dosages of 50 × 10^6^ DCs; twice intravenous and once intradermal. Three out of nine patients showed a partial response in the first 8 weeks. Two of these patients were treated shortly before start of DC treatment with chemotherapy. This might intervene with the result (18).

The second study published in 2016 (19), the same type of DCs were administered; this time in combination with cyclophosphamide, a drug inhibiting regulatory T-cells (20). Five postsurgical and 5 non-surgical MPM patients were treated. In one of the non-surgical patients, a partial response was found. Overall, 7 out of 10 patients lived longer than 24 months. The OS was promising with a mean survival of 37 months (19).

Since the process of obtaining proper autologous tumor cell lysates is very time consuming and patient reluctant to multiple pleural biopsies, an alternative source of antigens to pulse the DCs was investigated. DCs were pulsed by a spectrum of tumor associated antigens derived from allogeneic tumor lysate form human mesothelioma cell line cultures. These DCs were tested in 9 MPM patients including 5 subjects pretreated by chemotherapy. In these 9 patients, a partial response was established in 2 patients; one treatment-naïve patient and one pretreated patient, lasting 15 and 21 months. Disease control was described in all other patients, with a median overall survival higher than 22.8 months (21). To validate these promising results, a European (H2020) randomized phase II/III trial (DENIM) assessing DCs immunotherapy vs. best supportive care as maintenance treatment after standard first line chemotherapy is ongoing.

## Biomarkers

Similar to NSCLC, melanoma and other cancers, biomarkers to predict the response (or toxicity) to treatment in patients, are a crucial issue. In MPM, PD-L1 is expressed in 40–60% of the tumors, mostly in patients with sarcomatoid histology. PD-L1 expression is a negative prognostic factor for overall response to standard care but not for PFS or OS. In a retrospective study, the PD-L1 positive patients exhibited a mOS of 5 months, while median survival in PD-L1 negative patients was 14.5 months (22), while other studies and trials results had discrepancies on this finding (23).

In several studies, PD-L1 expression was correlated with response to PD-L1 inhibitors, with or without CTLA-4 inhibitors. In the PD(L)-1 monotherapy (2–6) studies responses to PD-L1 >1% varied between 19 and 44%. Generally, PD-L1 negative tumors show responses up to 10%, with only one study reporting an ORR of 56%; although in a small group of 9 patients (5). In the studies combining PD-(L)1 inhibitors with CTLA-4 inhibitors, a correlation between response and PD-L1 positive expression on tumors was found. In these studies (7, 8, 13) PD-L1 > 1% showed a response rate of 23–73%. Patients with PD-L1 negative tumors showed an ORR of 27–33%. Interestingly, the study of Scherpereel et al. (8) showed that the PD-L1 negative tumors had a similar response compared to the PD-L1 positive tumors to the combination therapy.

A reason for PD-L1 IHC not to be a very reliable biomarker might be the immune environment of MPM. In multiple studies a relatively low number of CD8^+^ tumor infiltrating lymphocytes (TIL) have been observed (24, 25). MPM is also known to have an increased suppressive immune environment, with a high amount of CD4^+^, FOXP3, and CD25^+^RO^+^ TILs. Marcq et al. showed in MPM with low numbers of CD8^+^TILs, that their function was either moderately or severely suppressed (26). A high number of CD8+ TILs on the other hand correlates with more tumor cell apoptosis, lower N-stage and higher overall survival (25, 27, 28). Higher numbers of PD-L1^+^CD8^+^TIL were found in sarcomatoid subtypes (26), which might explain the slightly better results in PD-(L)1 checkpoint inhibitor therapy. High CD8^+^TILs is a prognostic biomarker (28), it is not clear if this can also be used as a predictive biomarker in checkpoint inhibitors.

CTLA-4 is expressed in a little more than half of the MPM tissues. In the study of Roncella et al. CTLA-4 expression was measured in tissue, serum and pleural effusion of 45 patients. CTLA-4 expression seems a favorable prognostic factor, but this was only statistically significant in pleural fluid with a dead-rate reduction of 60% when a cut-off at 67 pg/ml soluble CTLA-4 was applied. Whether a positive finding of CTLA-4 expression in MPM will have therapeutic implications has not been investigated yet (29).

In NSCLC, tumor mutational burden (TMB) is a suggested biomarker to predict the efficacy in immunotherapy, in particular for the ipilimumab-nivolumab combination. As MPM harbor a low average TMB (30), this is thought to be of little prognostic use. One of the newer findings indicate that chromothripsis; which is chromosome scattering followed by random chromosome rearrangement, occurs more often in MPM and cannot be identified with whole genome sequencing. It is believed that the large parts of spliced DNA will accumulate in the cytoplasm and give rise to neoantigens (31).

Other factors that might correlate with response to checkpoint inhibitors such as HLA class I genotype, foregut microbiome composition are investigated but no results were reported yet (32).

## Discussion

The NCCN guidelines (2018) recommend nivolumab ± ipilimumab or pembrolizumab as subsequent systemic therapy (33). Most of the previous trials in MPM with immunotherapy show activity in a limited number of patients with low and manageable toxicity. As summarized in Table 1, the studies exhibited a large variation in outcome as measured by PFS and OS. This might be related to the relatively small size of most studies, and variations in pathology and study execution. These factors are possibly due to a patient selection bias, with different inclusion criteria (34). The only reported randomized trial, the PROMISE-meso trial, did show that pembrolizumab was not superior to chemotherapy in the second line in terms of PFS. Patients in both arms could cross-over to either pembrolizumab or chemotherapy after progression. It could imply that in daily practice both pembrolizumab and chemotherapy are effective, in selected groups of patients.

Response assessment in MPM is challenging. Modified RECIST (mRECIST) for pleural mesothelioma was developed in 2004. Recently, immune-based therapeutics (iRECIST) was published to stage solid tumors. In the previous described studies different RECIST criteria were used. This can be an explanation for the wide range in reported response rates (see Table 1). NIBIT-MESO used immune-related objective response (complete response or partial response) according to immune-related modified RECIST criteria in patients with pleural mesothelioma. They pointed out the importance of criteria for follow up. irRECIST is based on solid tumors, but does not take specific MPM response considerations into account. Therefor mRECIST 1.1 recommends adoption of irRECIST into mRECIST (35). More research is needed to assess the immune-related modified RECIST criteria.

Disease control rate (DCR) is a commonly used endpoint in MPM. However, this endpoint is subject to several forms of bias; the time points for DCR is inconsequent between studies. The DETERMINE trial measured DCR at ≥6 weeks after randomization (29%) (31), the KEYNOTE-028 reported DCR at 8 weeks (72%) (1), several studies at 12 weeks (5, 7, 8, 31)[38–67] while other studies did not specify at which time point DCR was measured (47–68%) (2–4, 9) (see Table 1). This leads to a time-to-event bias, making it hard to compare DCR between studies. By selecting the best patients, almost all small phase II trials recruit only performance status 0 or 1, there is a possibility that DCR is also a reflection of the tumor biology. We suggest that ORR is a better primary endpoint for future studies with immunotherapy in MPM, and reporting of the DCR as secondary endpoint at a pre specified time point.

The MAPS2 trial reported hyper progressive disease (HPD) due to immunotherapy, which raises questions. It was not reported how hyper progressive disease was measured. It is unclear if patients had 2 CT-scans without treatment before start of study-treatment, to be able to evaluate the growth rate. The subgroups were very small, ranging from 2 to 11 patients, and the relation between HPD and OS was not equal between the different definitions of HPD (17). It is not known if HPD is unique for immunotherapy. In the PROMISE-meso trial, also patients in the chemotherapy arm had an increase of up to 80% in tumor size at the first response evaluation (14).

To be able to distinguish which patient will benefit from immunotherapy and who will not, better biomarkers are urgently needed. As in NSCLC, PD-L1 positive patients, especially the non-epithelioid group, seem to have a better outcome compared to PD-L1 negative patients. Unfortunately, there is no validated clear-cut for the percentage of PD-L1 positive tumor cells, probably due to the heterogeneity of the tumor and other immunosuppressive and –activating factors such as tumor infiltrating lymphocytes, T-regs, inflammation, HLA class genotype, and microbiome composition. The need for better biomarkers is also high, to prevent costs and possible unnecessary complications due to immunotherapy.

Since malignant mesothelioma is a rare disease, selecting agents for large phase III trials should be based on impressive response rates of single agent phase II data and positive randomized phase II results. However, in MPM numbers of large phase II/III trials have been initiated based on very limited evidence; (e.g., the DETERMINE trial, the NVALT5 trial (thalidomide vs. best supportive care), the NGR015 trial (investigator choice plus NGR-hTNF or placebo), the VANTAGE-014 trial (vorinostat vs. placebo) and the COMMAND trial [maintenance defactinib or placebo)] (12, 36–39). Recommended endpoint for future RCT's in MPM would be to confirm an overall survival benefit with an HR of ≤ 0.7 and a gain of ≥3 months without a statistically significantly in grade 3–4 toxicities to preserve quality of life (40).

Although all patients eventually will experience a recurrence after first line chemotherapy, the standard of care (platinum- pemetrexed therapy) is effective with response rates around 45%, a median PFS of up to 7.3 months and a OS up to 16 months (41, 42). Results of the DREAM- study should be placed in perspective with a response rate of 48% and a PFS of 6.9 months (13).

In conclusion, immunotherapy seems to bring hope for a selected group of MPM patients but several crucial questions remain unanswered to date. Phase III randomized trials with clear primary end-points are on their way and will probably establish the role of immunotherapy in MPM. In addition, there is an urgent need for biomarkers to select the optimal candidates for immunotherapy among MPM patients in terms of efficacy and tolerance.

## Author Contributions

CG and FB performed a literature search, interpreted data, and wrote the manuscript. AS and PB supervised and contributed to the writing process.

### Conflict of Interest

PB research support and advisory function for MSD, BMS, Roche, Pfizer. AS participated to some expert boards, and was invited to recent WCLC and ASCO annual meetings with Astra-Zeneca, BMS, MSD and Roche. The remaining authors declare that the research was conducted in the absence of any commercial or financial relationships that could be construed as a potential conflict of interest.
